# An Original Stepwise Multilevel Logistic Regression Analysis of Discriminatory Accuracy: The Case of Neighbourhoods and Health

**DOI:** 10.1371/journal.pone.0153778

**Published:** 2016-04-27

**Authors:** Juan Merlo, Philippe Wagner, Nermin Ghith, George Leckie

**Affiliations:** 1 Unit for Social Epidemiology, Faculty of Medicine, Lund University, Malmö, Sweden; 2 Centre for Clinical Research Västmanland, Uppsala University, Uppsala, Sweden; 3 Research Unit of Chronic Conditions, Bispebjerg University Hospital, Copenhagen, Denmark; 4 Centre for Multilevel Modelling, University of Bristol, Bristol, United Kingdom; Utrecht University, NETHERLANDS

## Abstract

**Background and Aim:**

Many multilevel logistic regression analyses of “neighbourhood and health” focus on interpreting measures of associations (e.g., odds ratio, OR). In contrast, multilevel analysis of variance is rarely considered. We propose an original stepwise analytical approach that distinguishes between “specific” (measures of association) and “general” (measures of variance) contextual effects. Performing two empirical examples we illustrate the methodology, interpret the results and discuss the implications of this kind of analysis in public health.

**Methods:**

We analyse 43,291 individuals residing in 218 neighbourhoods in the city of Malmö, Sweden in 2006. We study two individual outcomes (psychotropic drug use and choice of private vs. public general practitioner, GP) for which the relative importance of neighbourhood as a source of individual variation differs substantially. In Step 1 of the analysis, we evaluate the OR and the area under the receiver operating characteristic (AUC) curve for individual-level covariates (i.e., age, sex and individual low income). In Step 2, we assess general contextual effects using the AUC. Finally, in Step 3 the OR for a specific neighbourhood characteristic (i.e., neighbourhood income) is interpreted jointly with the proportional change in variance (i.e., PCV) and the proportion of ORs in the opposite direction (POOR) statistics.

**Results:**

For both outcomes, information on individual characteristics (Step 1) provide a low discriminatory accuracy (AUC = 0.616 for psychotropic drugs; = 0.600 for choosing a private GP). Accounting for neighbourhood of residence (Step 2) only improved the AUC for choosing a private GP (+0.295 units). High neighbourhood income (Step 3) was strongly associated to choosing a private GP (OR = 3.50) but the PCV was only 11% and the POOR 33%.

**Conclusion:**

Applying an innovative stepwise multilevel analysis, we observed that, in Malmö, the neighbourhood context per se had a negligible influence on individual use of psychotropic drugs, but appears to strongly condition individual choice of a private GP. However, the latter was only modestly explained by the socioeconomic circumstances of the neighbourhoods. Our analyses are based on real data and provide useful information for understanding neighbourhood level influences in general and on individual use of psychotropic drugs and choice of GP in particular. However, our primary aim is to illustrate how to perform and interpret a multilevel analysis of individual heterogeneity in social epidemiology and public health. Our study shows that neighbourhood “effects” are not properly quantified by reporting differences between neighbourhood averages but rather by measuring the share of the individual heterogeneity that exists at the neighbourhood level.

## Introduction

An established area of research in social epidemiology and public health concerns the investigation of “neighbourhood and health” and multilevel logistic regression analyses are frequently conducted for this purpose [1, 2] [3, 4] [5] [6]. Interest within such studies typically lies in estimating and interpreting measures of associations (e.g., the exponentiated regression coefficients or odds ratios, OR) between specific contextual characteristics and binary measures of individual health outcomes. In other settings, researchers routinely perform analyses of small area variation which, in their simplest form, are displayed as health league tables, “heat” or choropleth maps, or atlases of geographical variation. A common denominator in all these studies is that they analyse differences between group averages. For instance, the average risk of dying among individuals living in poor neighbourhoods might be compared to the average risk of dying among individuals living in rich neighbourhoods. Alternatively, statistics like indices of small area variation might be calculated to summarize the overall range or variation in group averages. All these studies disregard within-group individual-level variation in health outcomes except to estimate the statistical uncertainty around the estimated differences between group averages

In contrast, other researchers have explicitly concluded that we need to consider both differences between group averages and differences between individuals around these averages. In fact, information on individual-level variance in multilevel regression analysis provides indispensable information for understanding contextual influences on health [1–13] From this perspective, knowing the proportions of overall variation in health outcomes which are attributable to the contextual-level (e.g., the neighbourhood) is of fundamental relevance for operationalizing contextual phenomena and for identifying the relevant levels of analysis [3, 7, 11, 14–18]. This concept is rather intuitive when we think about the analogy between individual and collective bodies [7]. Also, using Rose’s terminology [19], in order to identify sick populations the simple quantification of differences between population averages of some health indicator is not appropriate. Rather, we need information on both population averages and the distribution of individual values around these averages. Through doing so, we are able to learn the share of the total outcome variance that is between population level averages so the larger this proportion, the more relevant the population level of analysis is [11]. This idea corresponds well with the notion of variance partition coefficients (VPC) and the concept of clustering as measured by intraclass correlation coefficients (ICC) [16].

Considering these ideas, we can identify at least three different analytical approaches in social epidemiology, all of which are dedicated to the investigation of contextual influences on binary measures of individual health.

The *small area variation approach* focuses on the analysis of geographic variance using aggregated geographical data often on small areas or zones at different spatial scales [20].

The *multilevel analysis of associations approach* performs multilevel logistic regression analysis or similar techniques to identify average associations (e.g., ORs) between specific contextual level variables and individual health adjusting for neighbourhood clustering [21],.

Finally, the *multilevel analysis of individual heterogeneity approach* combines both the multilevel analysis of associations for estimation of specific contextual effects and the multilevel analysis of variance (e.g., the degree of clustering, ICC) for the investigation of general contextual effects (i.e., non-specific contextual influences on health) [7] [4].

The *small area variation approach* typically applied in Public Health represents a refinement of classical ecological studies on aggregated data. The *multilevel analysis of associations approach* follows the conventional approach in probabilistic risk factors epidemiology [22], while the *multilevel analysis of individual heterogeneity approach* adopts a multilevel perspective for understanding heterogeneity of individual responses around the average risk in a group [2]. It is this last approach which we develop and promote in this study.

Interestingly, in spite of their independent origins and areas of application, the *multilevel analysis of individual heterogeneity approach* has many analogies with that adopted in other fields of epidemiology concerned with the identification of new candidate risk factors and biomarkers and the evaluation of diagnostic and screening test. In those research fields, it is well known that measures of average association like ORs provide limited information for gauging the performance of a diagnostic, prognostic, or screening marker [23]. Accordingly, the rule is that measures of association need be interpreted together with measures of discriminatory accuracy such, the area under the receiver operating characteristic curve (AUC) [24, 25]. Analogously, the *multilevel analysis of individual heterogeneity approach* argues that estimates of specific contextual effects (i.e., average measures of association) provide insufficient information if they are not accompanied by measures of general contextual effects (i.e., degree of clustering) [2–4].

In the *multilevel analysis of individual heterogeneity approach* the ICC for hierarchical multilevel structures [26] is a fundamental measure for quantifying general contextual effects. As a concept, the ICC (i.e., the share of the total outcome variance which lies at the context level, having adjusted for any covariates) is rather intuitive for continuous responses since the individual- and contextual-level variances are both estimated and defined on the same scale. However, the ICC proves less straightforward to understand and calculate when analysing binary responses via multilevel logistic regression because only the contextual-level variance is estimated. Furthermore, this variance is defined on the log-odds scale, rather than the binary response scale [26]. Nevertheless, a range of procedures for calculating the ICC for binary responses have been proposed, including a normal response approximation, the simulation method, and the Taylor series linearization, [14, 26–28]. However, it is the ICC based on the latent response formulation of the model which has become most widely adopted. No doubt partly due to these complications, a range of alternatives to the ICC for binary responses have also been proposed to quantify the extent of general contextual effects. These include the pairwise odds ratio (PWOR)[18] and measures of heterogeneity such as the median odds ratio (MOR)[29, 30]. In any case, it is important to realize that the ICC is itself a measure of discriminatory accuracy [31, 32]. Therefore, taking advantage of the analogy between the concept of discriminatory accuracy and the notion of general contextual effects, a simple but innovative approach is to express general contextual effects by means of measures of discriminatory accuracy like the AUC [33, 34]. The AUC measure is well established among epidemiologists, public health practitioners and physicians and its computation is straightforward using standard statistical software.

In the current study, we present a novel three-step approach for the systematic investigation of observational multilevel (e.g., individual and neighbourhood) effects on binary measures of individual health and health care utilization, distinguishing between specific and general contextual effects. To make our approach as accessible as possible, we present a conceptual and didactic treatment of the issues rather than a technical and mathematical one. We introduce and then demonstrate the utility of AUC as a measure of general contextual effects and we compare it to the ICC and the MOR. We illustrate our approach by analysing two different binary outcomes: (i) use of psychotropic medication, which is related to both psychological health and access to medication; and (ii) individual choice of a private vs. a public general practitioner (GP), which is a behavioural outcome.

## Population and methods

### Study sample

We drew our sample of individuals from the LOMAS (Longitudinal Multilevel Analysis in Scania) database containing fully anonymised data on all individuals living in the county of Scania, Sweden during the years 1968–2006. The database includes geographic, demographic and socioeconomic information on all individuals as well as data on their health care and medication use [35]. The sample consists of all individuals aged 35–64 years residing in the city of Malmö on 31st December 2005 (N = 99,266), who were still alive on 31st December 2006 (N = 98,536). We further restricted this sample to those with at least one contact with primary health care during the year 2006 (N = 46,675) as well as residing in neighbourhoods with at least 30 people who fulfilled the same selection criteria (N = 43,588). The reason for the latter was to further ensure the anonymity of the database. Otherwise, the size of the neighbourhood is not a problem for performing multilevel regression analysis as small neighbourhoods provide uncertain information and are shrunken towards the overall mean in the multilevel regression [16]. Lastly, we dropped 297 (0.7%) individuals who had missing values for individual income. The final study sample consisted of 43,291 individuals within 218 neighbourhoods.

Our study is representative for those individuals with at least a one contact with primary health care and this condition is necessary to investigate the choice of a private versus a public GP. However, in the case of psychotropic drug use we could have studied the whole sample of people 35–64 year old. Therefore, strictly our analysis concerns use of psychotropic drugs among primary health care patients rather than the whole population.

The National Board of Health and Welfare and Statistics Sweden constructed the initial LOMAS database by means of record linkage of different registers using the unique Swedish personal identification number. Finally, the Swedish authorities delivered the LOMAS database to us without the personal identification numbers to ensure the anonymity of the subjects and after aprobal from The Regional Ethics Review Board in southern Sweden as well as the data safety committees from the National Board of Health and Welfare and from Statistics Sweden.

For the purpose of our study we used the LOMAS database to create a fully anonymized sample that besides the exclusion criteria contained only information on gender, age in 5-year groups, as well as individual and neighbourhood income in two categories. We also deleted and replaced the identification codes of the neigbourhoods and the LOMAS codes of the individuals. This fully anonymized sample provides sufficient information to replicate our analyses but completely prevent the identification or specific individual or neighbourhoods. This sample is provided in the Online Supplementary Materials in MLwiN format (S1 Data), SPSS format (S2 Data) and Stata format (S3 Data).

### Assessment of variables

#### Outcome variables

To illustrate our three-step approach, we carried out two empirical analyses. In the first analysis the outcome variable was defined as use (= 1) or not (= 0) of psychotropic medication during 2006. We defined psychotropic medication as Anatomical Therapeutic Chemical (ATC) Classification System [36] codes N05B (Anxiolytics), N05C (Hypnotics and sedatives) and N06A (Antidepressants). In the second analysis, the response variable was whether a person had visited a private (= 1) or public (= 0) specialist physician in a general practice (GP) during the year.

### Individual characteristics

In order to illustrate our approach as clearly as possible, we considered only three individual-level covariates: age categorized into six age groups, 35–39, 40–44, 45–49, 50–54, 55–59, and 60–64 years, using the youngest age group as the reference category in the model specifications; sex that compared men (= 1) with women (= 0); and income categorized as ‘low’ when having less that the median income in Malmö, or ‘high’ otherwise. In the analysis of psychotropic medication the reference category was high income while in the analysis of private GP choice the reference category was low income. These choices are cosmetic, but ensure that we estimate positive rather than negative associations between the outcome and income which are easier for readers to interpret (psychotropic medication use is higher among the poor while private GP use is higher among the rich). The median income in Malmö was derived from individualized household disposable income in 2004 for all individuals aged 35 to 85 in the city.

### Neighbourhood variables

We defined *neighbourhoods* using small-area market statistics (SAMS) boundaries created by Statistics Sweden [37]. The SAMS boundaries are based on municipalities’ sub-division boundaries which are constructed to maximise the internal homogeneity of housing tenure. The resulting neighbourhoods have an average population of around 1000 individuals.

For simplicity, we categorized neighbourhoods as ‘rich’ or ‘poor’ according to whether the proportion of low income individuals in each neighbourhood was below the median across all neighbourhoods in the city. Paralleling the way we entered individual income into our models, in the analysis of psychotropic medication use we set the reference category for neighbourhood income to be rich neighbourhoods while in the analysis of private GP use we set the reference category to be poor neighbourhoods.

### Multilevel analysis of heterogeneity

The data have a two-level hierarchical structure with individuals (level 1) nested within neighbourhoods (level 2). For the analysis we applied a three step-approach consisting of fitting, interpreting and contrasting the results of three consecutive two-level logistic regression models: the individual effects model (Step 1); the general contextual effects model (Step 2); and the specific contextual effects model (Step 3).

Let *y*_*ij*_ denote the binary response of interest (e.g., use of psychotropic medication or private GP) for individual *i* (*i* = 1,…,*n*_*j*_) in neighbourhood *j* (*j* = 1,…,*J*).

#### Step 1—The individual effects model

Step 1 simply consists of fitting a conventional single-level logistic regression for *y*_*ij*_ including only the individual-level covariates; neighbourhoods are completely ignored. In terms of our two illustrative applications, the covariates are age, sex and income. See also the MLwiN worksheets for fitting this model in supplementary information (S1 and S4 Models). The model is therefore written as

$$y_{ij}\;\sim \;\text{Binomial}\left(1,\;\pi_{ij}\right),$$ (1)

$$\text{logit}\left(\pi_{\text{ij}}\right)=\beta_{0}+\beta_{1}\text{ag}{\text{e}}_{\text{ij}}+\beta_{2}\text{se}{\text{x}}_{\text{ij}}+\beta_{3}\text{incom}{\text{e}}_{\text{ij}},$$ (2)

where *π*_*ij*_ denotes the probability that individual *i* in neighbourhood *j* uses psychotropic medication (or private GP) given their individual characteristics age_*ij*_, sex_*ij*_ and income_*ij*_.

The regression coefficients *β*_1_, *β*_2,_ *β*_3_ measure the associations between the log-odds of the health outcome and each covariate all else equal and when exponentiated these are translated to ORs. For ease of illustration we have entered age into the model linearly, but we shall relax this assumption when we fit the model. Post-estimation, predicted probabilities ${\hat{\pi }}_{ij}$ are calculated for each individual and are used to calculate the AUC for the model.

The AUC [33, 34] is constructed by plotting the true positive fraction (TPF) (i.e., sensitivity) against the false positive fraction (FPF) (i.e., 1 − specificity) for different binary classification thresholds of the predicted probabilities. The AUC measures the ability of the model to correctly classify individuals with or without the outcome (e.g., using or not psychotropic medication or visiting a private vs. a public GP) as a function of individuals’ predicted probabilities. The AUC takes a value between 1 and 0.5 where 1 is perfect discrimination and 0.5 would be as equally as informative as flipping a coin [23] (i.e., the covariates have no predictive power). The AUC of the Step 1 model quantifies the accuracy of using individual-level information alone for identifying individuals with the outcome.

There are alternative measures of discriminatory accuracy like net reclassification improvement (NRI) and the integrated discrimination improvement (IDI) [38–40]. However, the NRI and IDI depend on the calibration of the models and are non-proper evaluation metrics so the use of these measures is not recommendable [41].

#### Step 2 –The general contextual effects model

Step 2 consists of extending the Step 1 model from a conventional single-level logistic regression model to a two-level individuals-within-neighbourhoods logistic regression model. See also the MLwiN worksheets fitting this model in supplementary information (S2 and S5 Models). This extended model is written as

$$y_{ij}\;\sim \;Binomial\left(1,\pi_{ij}\right),$$ (3)

$$\text{logit}\left(\pi_{\text{ij}}\right)=\beta_{0}+\beta_{1}{\text{age}}_{\text{ij}}+\beta_{2}{\text{sex}}_{\text{ij}}+\beta_{3}{\text{income}}_{\text{ij}}+{\text{u}}_{\text{j}},$$ (4)

$$u_{j}\sim N\left(0,\sigma_{u}^{2}\right),$$ (5)

where *u*_*j*_ denotes the random effect for neighbourhood *j*. These effects are assumed normally distributed with zero mean and variance $\sigma_{u}^{2}$, a parameter to be estimated.

Postestimation, values can be assigned to these effects via empirical Bayes prediction. These predictions ${\hat{u}}_{j}$ are sometimes referred to as shrinkage estimates as their values are shrunk towards the population-average of zero by a shrinkage factor proportional to the amount of information available on each neighbourhood (essentially the neighbourhood size). Shrinkage is desirable as it protects one against over interpreting the otherwise often extreme predictions associated with very small neighbourhoods. The statistical uncertainty surrounding these predictions can also be calculated and communicated via error bars (e.g., 95% confidence intervals). This uncertainty must be taken into account when ranking neighbourhoods, for example by predicted prevalence of the health outcome, as such rankings have been shown to be especially unreliable (see elsewhere for an extended explanation and empirical examples) [42–44]. More generally, the interpretation of neighbourhood rankings needs be done in relation to the *general contextual effect* (see elsewhere for empirical examples) [4].

The *general contextual effect* is appraised using the estimated between-neighbourhood variance ${\hat{\sigma }}_{u}^{2}$ as this quantifies the variability in unobserved influences on the health outcome common to individuals living in in the same neighbourhood. Thus, ${\hat{\sigma }}_{u}^{2}$ is assumed to reflect variation in any direct effects of neighbourhood context captured by the neighbourhood boundaries (i.e., “causal” effect of place). However, in an observational study, it might also reflect neighbourhood compositional differences in unmodelled individual characteristics (e.g., unobserved selection of individuals into neighbourhoods). We calculated three different measures of *general contextual effects*: (i) the change in the AUC compared with the Step 1 model; (ii) the ICC; and (iii) the MOR.

1. While the AUC of the Step 1 model quantifies the accuracy of using individual-level information alone for identifying individuals with, or without the outcome, the predicted probabilities from the Step 2 model are based on both the individual-level covariates and the predicted neighbourhood random effect ${\hat{u}}_{j}$. Consequently, the AUC of the Step 2 model can be compared with that from Step 1 to quantify the added value of having information on the neighbourhood of one’s residence when it comes to identifying the outcome of the individuals. Therefore, in this approach the general contextual effect of the neighbourhood is appraised by quantifying the increase in the AUC achieved when adding general neighbourhood information to the individual level predictions calculated in the Step 1 model. The larger this difference, the greater the general neighbourhood effect is.
2. We chose to calculate the ICC based on the latent response formulation of the model as it is the approach most widely adopted in applied work. This formulation assumes a latent continuous response underlies the observed binary response and it is this latent response for which the ICC is calculated and interpreted. The higher the ICC, the more relevant neighbourhood context is for understanding individual latent response variation [14, 16, 26]. The ICC is calculated as
  $$\rho =\frac{\sigma_{u}^{2}}{\sigma_{u}^{2}+\frac{\pi^{2}}{3}}$$ (6)
  where $\frac{\pi^{2}}{3}$ denotes the variance of a standard logistic distribution. (Note that here *π* denotes the mathematical constant 3.1416…, not the probability.)
3. The MOR [14, 29, 30] is an alternative way of interpreting the magnitude of the neighbourhood variance. The MOR translates the neighbourhood variance estimated on the log-odds scale, to the widely used OR scale. This makes the MOR comparable with the OR of individual and neighbourhood covariates. The MOR is defined as the median value of the distribution of ORs obtained when randomly picking two individuals with the same covariate values from two different neighbourhoods, and comparing the one from the higher risk neighbourhood to the one from the lower risk neighbourhood. In simple terms, the MOR can be interpreted as the median increased odds of reporting the outcome if an individual moves to another neighbourhood with higher risk. Therefore, the higher the MOR the greater the general contextual effect. The MOR is calculated as
  $$MOR=exp\left(\sqrt{2\sigma_{u}^{2}}\Phi^{-1}\left(0.75\right)\right),$$ (7)
  where Φ^−1^(∙) represents the inverse cumulative standard normal distribution function. In absence of neighbourhood variation (i.e., $\sigma_{u}^{2}=0$), the MOR is equal to 1.

#### Step 3 –The specific contextual effects model

Step 3 consists of adding the neighbourhood covariate of interest to the model in order to estimate the specific OR for a contextual variable. In our case we are interested in the effect of neighbourhood income (i.e., rich or poor) on each outcome. See also the MLwiN worksheet for fitting this model in supplementary information (S3 and S6 Models). The step 3 model can be written as

$$y_{ij}\sim \text{Binomial}\left(1,\pi_{ij}\right),$$ (8)

$$\text{logit}\left(\pi_{ij}\right)=\;\beta_{0}+\beta_{1}{\text{age}}_{ij}+\beta_{2}{\text{sex}}_{ij}+\beta_{3}{\text{income}}_{ij}+\beta_{4}{\text{nincome}}_{j}+u_{j},$$ (9)

$$u_{j}\sim N\left(0,\sigma_{u}^{2}\right),$$ (10)

where nincome denotes the additional neighbourhood covariate.

Specific contextual effects measure the associations between contextual characteristics of the neighbourhood (e.g., rich or poor neighbourhood) and the individual outcome. As in the case of individual-level observational effects, specific contextual effects are estimated using measures of average effect such as ORs. However, an extended misunderstanding when applying multilevel regression analyses is to give a “population average” interpretation to the OR of contextual variables [14, 29, 30].

The point is that the multilevel regression provides regression coefficients for individual variables that are adjusted for the neighbourhood-level random effects. That is, they reflect the association between individual level variables and the outcome within a specific neighbourhood. They are therefore termed ‘‘neighbourhood specific” or ‘‘cluster specific” ORs. However, in multilevel logistic regression, a contextual OR can hardly be interpreted in this way since the contextual variable is constant for all individuals in the neighbourhood. The contextual OR can at best be interpreted as contrasting two neighbourhoods differing in the value of the contextual variable by one-unit, but which have identical value for the neighbourhood-level random effects (and all other covariates). To avoid this difficult interpretation, Larsen *et al* [29, 30] proposed the use of the 80% interval odds ratio (IOR-80%) as a way of including the neighborhood variance in the quantification of a contextual OR.

The lower and upper bounds of the IOR-80% for nincome are calculated as

$$exp\left(\beta_{4}\pm \sqrt{2\sigma_{u}^{2}}\Phi^{-1}\left(0.9\right)\right).$$ (11)

The IOR-80% is defined as the middle 80% range of the distribution of ORs formed by making random pairwise comparison between neighbourhoods exposed and non-exposed to the contextual variable. The IOR-80% interval is narrow if the between-neighbourhood variance $\sigma_{u}^{2}$ is small, and it is wide if the between-neighbourhood variance is large. If the IOR-80% interval contains 1, then for some neighbourhoods the association is in the opposite direction to the overall OR [29] [14].

An alternative to the IOR-80% is the Proportion of Opposed Odds Ratios (POOR). That is, the proportion of ORs with the opposite direction to the overall OR [14]. The values of the POOR extend between 0% and 50%. A POOR of 0% means all ORs have the same sign. A POOR of 50% means that half of the ORs are of the opposite sign and so the association is very heterogeneous. For our binary measure of neighbourhood income, the POOR is calculated as

$$POOR=\Phi \left(-\frac{\beta_{4}}{\sqrt{2\sigma_{u}^{2}}}\right).$$ (12)

Observe that in Step 2 we calculated the AUC as a way of quantifying neighbourhood general contextual effects. In Step 3, we included a specific contextual characteristic of the neighbourhood (i.e., low neighbourhood income) into the model in order to quantify specific contextual effects. However, adding this specific contextual variable cannot increase the AUC obtained in the Step 2 model since that model gives the maximum AUC that can be obtained by combining the available individual information and the neighbourhood identity. The latter captures the totality of potentially observable, but also unobservable neighbourhood factors. The inclusion of a specific neighbourhood contextual variable as a fixed-effect covariate will explain some of that neighbourhood variance (that is, decrease the average absolute size of the neighbourhood *u*_*j*_ estimates) and, thereby reducing the predictive role of the neighbourhood random effects. However, this change to the model specification simultaneously improves the model prediction through the addition of the regression coefficient for the neighbourhood income variable. Because of this balance the discriminatory accuracy of the Step 2 and 3 models will be effectively the same.

Step 3 provides a way of understanding the mechanism behind the observed general contextual effects. For this purpose we can calculate the proportional change in variance (PCV) defined as the proportion of the neighbourhood variance in Model 2 explained by adding the specific neighbourhood effect (i.e., neighbourhood income variable) in Model 3

$$PCV=\frac{\sigma_{u\left[Model\;2\right]}^{2}\;-\;\sigma_{u\left[Model\;3\right]}^{2}}{\sigma_{u\left[Model\;2\right]}^{2}}.$$ (13)

In our case, a large PCV would suggest that the general contextual effect is substantially mediated by the neighbourhood income variable.

See also the Stata do-file for running all the models (S1 Stata) and the Excel sheet (S1 Calculation Sheet) for the calculation of the MOR, 80% IOR, POOR, ICC and PCV in the supporting information files.

#### Summary of the multilevel analysis of heterogeneity approach

In multilevel analysis of heterogeneity, we need a joined up analysis that includes individual variables, neighbourhood boundaries, and neighbourhood characteristics. We need to include measures of association, variance and discriminatory accuracy. The simplistic “risk factor” approach based on the calculation of ORs alone is insufficient

In our two example studies we perform a series of three consecutive regression models.

We start with Model 1 (Step 1) that only includes individual-level covariates in a standard (i.e., single-level) logistic regression. The selection of these individual variables is based on the assumption that they condition the outcome and also the neighbourhood of residence. For instance, age is associated with use of psychotropic medicine and individuals may move to certain neighbourhoods when they become older. That is, we aim to prevent compositional confounding in later regression analyses. The candidate individual-level variables are not mediators of the neighbourhood effects. In our example the neighbourhood cannot change the age of the individuals. Besides the average ORs for the individual-level variables, the fundamental information in Model 1 is the size of the AUC.

In Model 2 (Step 2) we quantify the added value of having neighbourhood level information. We only include the neighbourhood boundaries without specifying any neighbourhood characteristic. We analyse the change in the AUC compared with Model 1. We also interpret the ICC and the MOR. This information tells us about the size of the general contextual effect.

In the final model, Model 3 (Step 3), we include specific neighbourhood information (neighbourhood income). In this model, the interpretation of the OR, the IOR and POOR must always be done in relation to the neighbourhood variance $\sigma_{u}^{2}$ of Model 2 and the PCV associated with moving from Model 2 to Model 3. For instance, suppose Model 2 estimated a high value for $\sigma_{u}^{2}$ and therefore a high ICC for the binary outcome “visiting a private vs. a public GP”. Thereafter, in Model 3, we include a contextual variable (neighbourhood high income). If neighbourhood high income is associated with the outcome (a high OR) and it explains a large share of $\sigma_{u}^{2}$ (PCV is high) the IOR-80% will be narrow and the POOR low. This case illustrates a situation where the neighbourhood context conditions the outcome (i.e., high $\sigma_{u}^{2}$ and ICC). It also demonstrates that this influence appears mediated by the contextual variable (neighbourhood high income) so the contextual variable is not only strongly associated with the outcome but it also explains the neighbourhood variance and thereby shows a narrow IOR-80% or a low POOR. In other words, the conclusion would be that the neighbourhood context influences the individual choice of GP and that this influence has to do with the socioeconomic circumstances of the neighbourhoods

However, there are other possible situations. For instance, $\sigma_{u}^{2}$ could be very low from the beginning (Model 2) and the contextual variable could be significantly associated with the outcome but still does not explain much of the $\sigma_{u}^{2}$ (i.e., low PCV) in Model 3. Nevertheless, since $\sigma_{u}^{2}$ was low from the beginning, the IOR-80% would also be narrow and the POOR low. In this case the neighbourhood context would have a small influence on the individual choice of GP even if the socioeconomic circumstances of the neighbourhoods are, on average, associated with the outcome and the IOR-80% is narrow.

#### Model estimation

The models were estimated using Markov chain Monte Carlo (MCMC) methods as implemented in the MLwiN multilevel modelling software [45]. We specify diffuse (vague, flat, or minimally informative) prior distributions for all parameters. We use quasilikelihood estimation to provide good starting values for all parameters. For each model, we specified a burn-in length of 5,000 iterations and a monitoring chain length of 10,000 iterations. Visual assessments of the parameter chains and standard MCMC convergence diagnostics suggest that the lengths of these periods are sufficient. The Bayesian deviance information criterion (DIC) was used as a measure of goodness of fit of our models [46]. The DIC considers both the model deviance and its complexity. Models with smaller DIC are preferred to models with larger DIC, with differences of five or more considered substantial [47].

Observe that we fitted the models by a fully Bayesian approach using MCMC. However, since the results are very similar, we simply predicted the neighbourhood random effects by maximum likelihood estimation (i.e., the empirical Bayes prediction). To be strict we should report the means of the MCMC chains for each predicted neighbourhood random effect. The fully Bayesian procedure is so far technically more complicated and time demanding but can be performed following the indication provided in the section 4.6 of the MLwiN MCMC manual [48]. The advanced reader may apply the fully Bayesian procedure using the databases supplied as complementary information.

### Ethics statement

The National Board of Health and Welfare and Statistics Sweden constructed the LOMAS database by means of record linkage of different registers using the unique Swedish personal identification number. Finally, the Swedish authorities delivered the LOMAS research database to us without the personal identification numbers to ensure the anonymity of the subjects. The Regional Ethics Review Board in southern Sweden as well as the data safety committees from the National Board of Health and Welfare and from Statistics Sweden approved the construction of the LOMAS database.

For the purpose of our study we used the LOMAS database to create a fully anonymized sample containing only information on gender, age in 5-year categories, as well as individual and neighbourhood income in two categories. We also deleted and replaced the identification codes of the neigbourhoods and the LOMAS codes of the individuals. This fully anonymized sample provides sufficient information to replicate our analyses but completely prevent the identification or specific individual or neighbourhoods. This sample is provided in the Online Supplementary Materials in SPSS format (S1 Data), Stata format (S2 Data) and MLwiN format (S3 Data).

## Results

### Characteristics of the population (Table 1)

**Table 1:** Characteristics of the population 35–65 year-olds in Malmö, 2006 by neighbourhood income.

	Neighbourhood	
	Poor	Rich
**Number of neighbourhoods**	93	125
**Number of individuals**	22780	20511
**Number of individuals in the neighbourhood (median, range)**	202 (36–910)	142 (47–592)
**Psychotropic drugs**	29%	23%
**Private GP**	11%	35%
**Low income**	60%	27%
**Men**	45%	42%
**Age (year-groups)**		
** 35–39**	19%	17%
** 40–44**	18%	17%
** 45–49**	17%	15%
** 50–54**	16%	16%
** 55–59**	16%	18%
** 60–64**	14%	17%

In the study sample, use of psychotropic drugs was more frequent in individuals with low income and in poor neighbourhoods while the opposite was true for visiting a private GP. Rich neighbourhoods had a higher percentage of people 55 years or older and a slightly lower percentage of men than poor neighbourhoods.

### Analysis of the use of psychotropic drugs (Table 2)

**Table 2:** Multilevel logistic regression analysis of psychotropic drug use in the 35–65 year-old population of Malmö, 2006. Values are odds ratios (OR) with 95% confidence interval (CI) unless stated otherwise. The intercept is not shown in the table.

	Simple logistic regression analysis	Multilevel logistic regression analysis	
	Model 1	Model 2	Model 3
**Specific Individual Average Effects**			
Men vs. women	0.61 (0.58–0.64)	0.60 (0.58–0.63)	0.60 (0.58–0.63)
Age groups			
35–39	Reference		
40–44	1.35 (1.24–1.46)	1.35 (1.25–1.45)	1.35 (1.24–1.46)
45–49	1.63 (1.50–1.77)	1.64 (1.51–1.77)	1.63 (1.51–1.77)
50–54	1.81 (1.67–1.95)	1.82 (1.69–1.96)	1.82 (1.68–1.97)
55–59	1.91 (1.76–2.07)	1.94 (1.80–2.10)	1.95 (1.81–2.10)
60–64	1.95 (1.80–2.11)	2.00 (1.85–2.16)	2.01 (1.86–2.17)
Low vs. high income	1.67 (1.60–1.74)	1.56 (1.49–1.64)	1.52 (1.44–1.59)
**Specific Contextual Average Effects**			
Low vs. high neighbourhood income			1.29 (1.21–1.38)
80% IOR			0.99–1.69
POOR (%)			11
**General contextual effects**			
Neighbourhood variance		0.038 (0.026–0.054)	0.022 (0.012–0.035)
PCV (%)			42
ICC (%)		1.1 (0.8–1.6)	0.7 (0.4–1.1)
MOR		1.20 (1.17–1.25)	1.16 (1.11–1.20)
AUC	0.616 (0.610–0.622)	0.630 (0.625–0.636)	0.629 (0.623–0.635)
AUC change*		0.014	-0.001
**Goodness of fit**			
DIC	48205	48063	48041
DIC change*		-142	-22

IOR: interval odds ratio. POOR: proportion of opposed odds ratios. PCV: proportional change in the variance. ICC: intra-class correlation coefficient. MOR: median odds ratio. AUC: area under the receiver operating characteristic curve. DIC: Bayesian deviance information criterion.

*: Change in relation to the previous model.

#### Specific Individual Average Observational Effects

**Fig 1:**
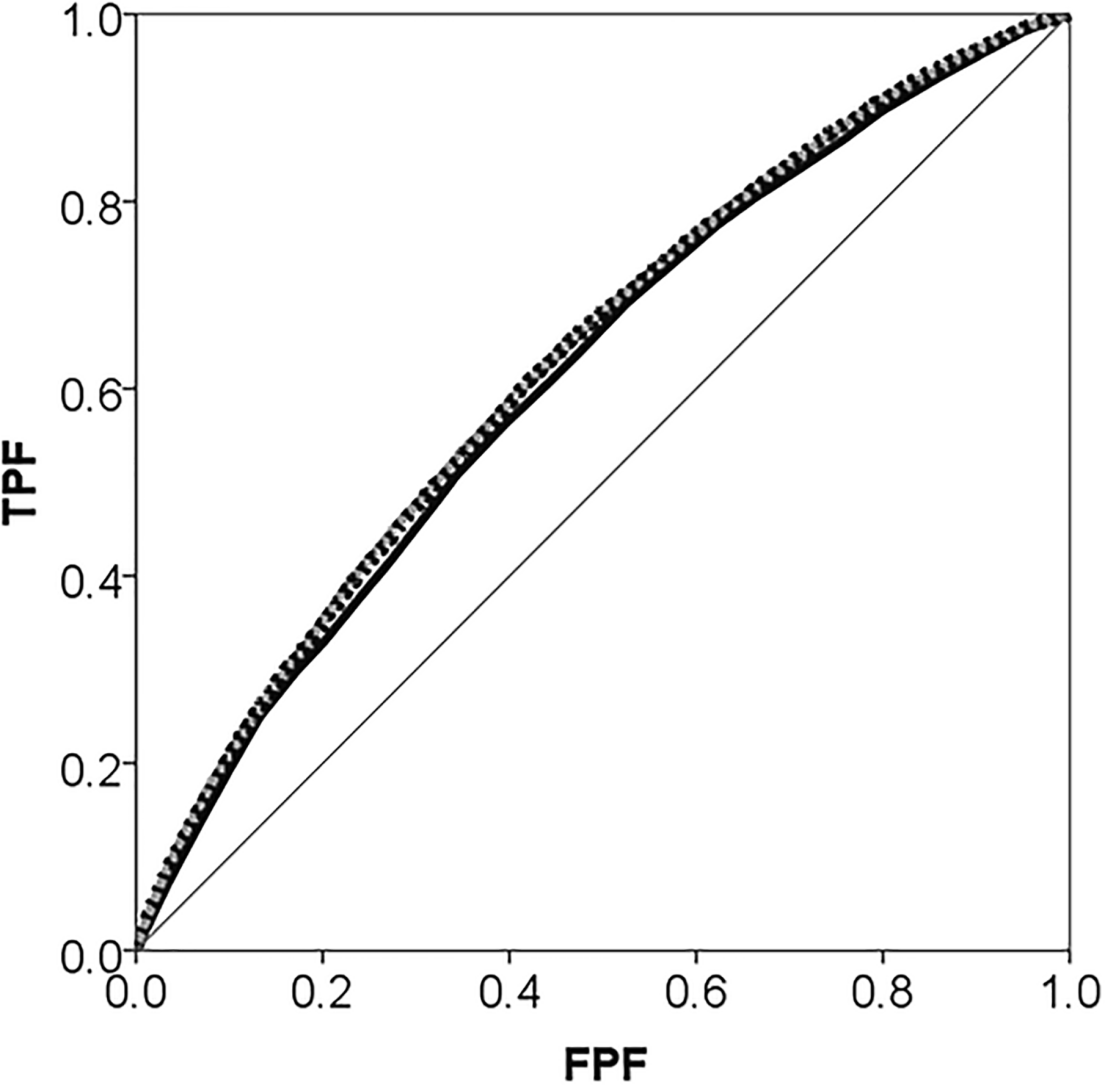
Areas under the receiver operating characteristic (AUC) curve for use of psychotropic drugs during 2006 in the city of Malmö, Sweden plotted separately for Model 1 which only adjusts for individual-level covariates age, sex and income (black thick line), and Model 2 which additionally adjust for neighbourhood of residence (grey dotted line). The diagonal line represents an AUC equal to 0.50.

The individual level population average Model 1 shows that use of psychotropic drugs increases monotonically with age and was more frequent for women and among people with low income. These individual characteristics, however, were not sufficient for predicting individuals’ use of psychotropic drugs with any degree of accuracy since the AUC was low (i.e., 0.616.) (Fig 1). In Model 2, the cluster specific association between individual income and use of psychotropic drugs was slightly lower than the population average association in Model 1.

#### Specific Contextual Average Observational Effects: IOR and POOR

In Model 3 we observed that, over and above individual income, age and sex, living in a low income neighbourhood conclusively increased the individual probability of use of psychotropic drugs (i.e., OR = 1.29). However, the 80%-IOR included 1 and the percentage of ORs of opposite direction was considerable (POOR = 11%).

#### General Contextual Observational Effects: neighbourhood variance, ICC, MOR and AUC

In Model 2, The ICC and the MOR were low (i.e., 1.1% and 1.20 respectively) which indicated that the neighbourhoods, as defined by the SAMS geographical boundaries, do not appear to capture a relevant context for understanding an individual’s propensity of using psychotropic drugs.

The added value of knowing an individual’s neighbourhood of residence besides individual information (age, sex and income) was very small since the AUC only increased 0.014 units when comparing Model 2 with Model 1 (Fig 1).

In Model 3, inclusion of the neighbourhood income variable explained 42% of the neighbourhood variance and decreased the ICC and MOR values to 0.7% and 1.16 respectively.

**Fig 2:**
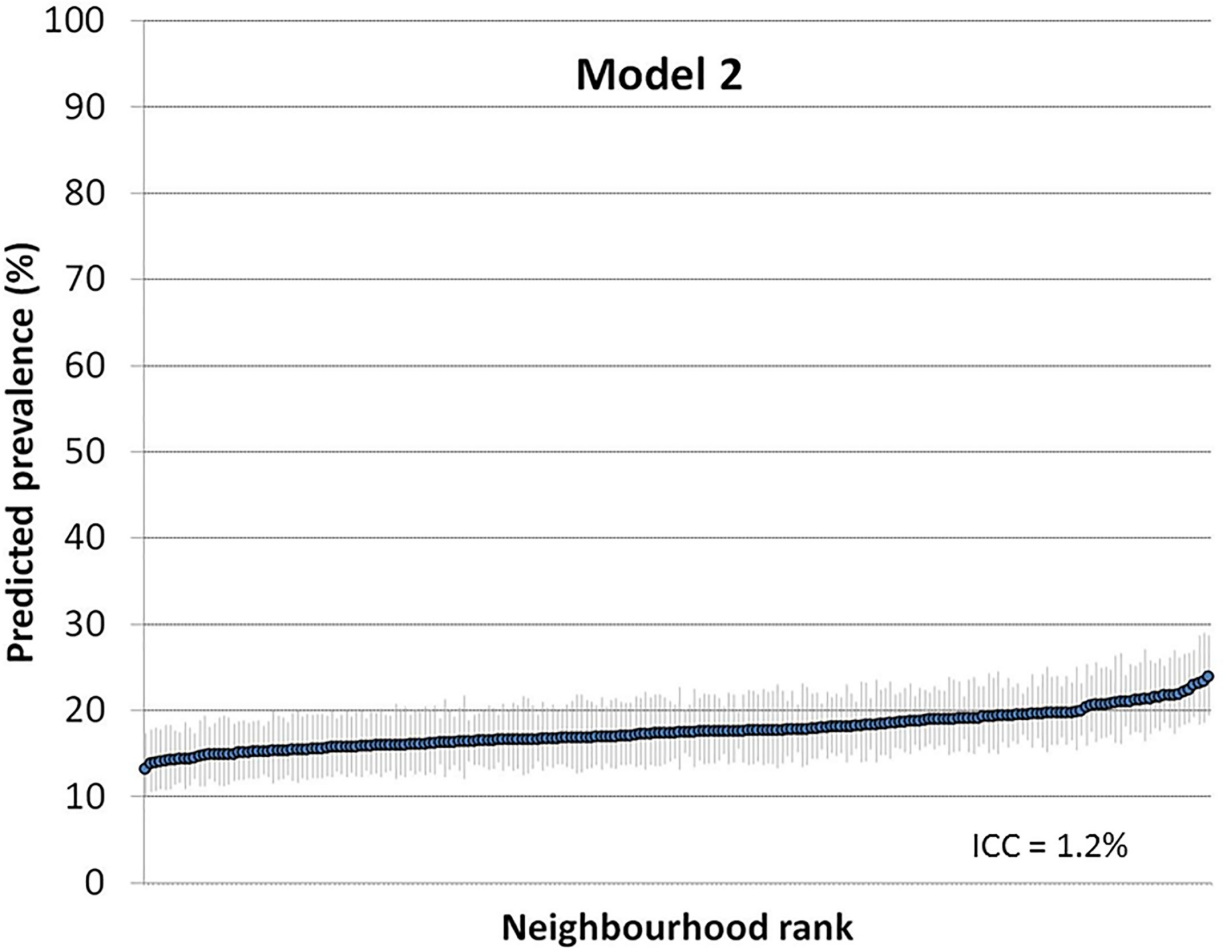
Predicted prevalence of psychotropic drug use in each neighborhood with 95% confidence intervals versus ranking. Predictions are for the reference individual in model 2, i.e.,female age 35–39, high income.

**Fig 3:**
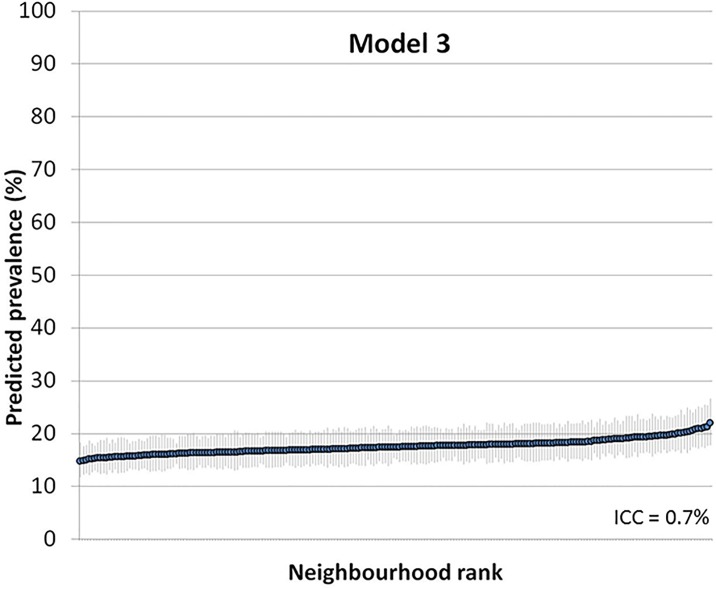
Predicted prevalence of psychotropic drug use in each neighborhood with 95% confidence intervals versus ranking. Predictions are for the reference individual in model 3, i.e., female, age 35–39, high income and living in a high income neighborhood (model 3).

Figs 2 and 3 show the ranking of the neighbourhoods of Malmö in 2006 according to their predicted prevalence of psychotropic drug use in each neighborhood with 95% confidence intervals versus ranking. Predictions are for the reference individual in model 2, i.e., female, age 35–39, high income (Fig 2), and for the reference individual in model 3, i.e., female, age 35–39, high income and living in a high income neighborhood (Fig 3).

Figs 2 and 3 indicates that there was considerable uncertainty in the ranking of the neighbourhoods, which expressed itself as a substantial overlapping of the confidence intervals. These “league tables” are only based on neighbourhood differences and need to be interpreted side-by-side with measures of general neighbourhood effects. Indeed, the ICC was very low in both models.

Analysis of choosing a private vs. a public specialist physician in general practice (Table 3)

**Table 3:** Multilevel logistic regression analysis of choosing a private versus a public specialist in the 35–65 year-old population of Malmö, 2006, Values are odds ratios (OR) and 95% confidence interval (CI) unless stated otherwise.

	Simple logistic regression analysis	Multilevel logistic regression analysis	
	Model 1	Model 2	Model 3
**Specific individual average effects**			
Men vs. women	0.96 (0.92–1.01)	0.94 (0.88–1.01)	0.94 (0.88–1.01)
Age groups			
35–39	Reference		
40–44	1.01 (0.93–1.09)	1.07 (0.94–1.20)	1.07 (0.94–1.20)
45–49	1.02 (0.94–1.11)	1.22 (1.07–1.37)	1.22 (1.07–1.37)
50–54	1.08 (1.00–1.17)	1.25 (1.10–1.41)	1.25 (1.10–1.41)
55–59	1.21 (1.12–1.31)	1.30 (1.16–1.46)	1.30 (1.16–1.46)
60–64	1.20 (1.10–1.30)	1.20 (1.06–1.35)	1.20 (1.06–1.35)
High vs. low income	2.13 (2.02–2.24)	1.14 (1.06–1.22)	1.13 (1.04–1.22)
**Specific contextual average effects**			
High vs. low neighbourhood income			3.50 (2.13–5.78)
80% IOR			0.09–130.28
POOR (%)			33
General contextual effects*			
Neighbourhood variance		4.479 (3.699–5.502)	3.980 (3.277–4.892)
PCV (%)			11
ICC (%)		57.8 (53.1–62.7)	54.9 (50.1–59.9)
MOR		7.53 (6.42–9.37)	6.71 (5.62–8.25)
AUC	0.600 (0.593–0.606)	0.895 (0.891–0.899)	0.895 (0.891–0.899)
AUC change*		0.295	0.000
**Goodness of fit**			
DIC	44726	24647	24648
DIC change*		-20079	1.28

IOR: interval odds ratio. POOR: proportion of opposed odds ratios. PCV: proportional change in the variance. ICC: intra-class correlation coefficient. MOR: median odds ratio. AUC: area under the receiver operating characteristic curve. DIC: Bayesian diagnostic information criterion.

*: change in relation to the previous model

#### Specific Individual Average Observational Effects

The population average Model 1 indicates that the odds of choosing a private GP were similar for men and women, and that they were somewhat higher among individuals aged 50 to 64 than among younger individuals. High individual income clearly increased the odds of choosing a private GP. These individual characteristics, however, were not sufficient for predicting individuals’ choice of GP with any degree of accuracy since the Model 1 AUC was low (i.e., 0.600).

Interestingly, the association between individual income and choosing a private GP declined when we recognized the multilevel structure of the data and included the neighbourhood level as a random effect in Model 2. This situation expresses the fact that the individual association in Model 1 was capturing not only a modest within neighbourhood association but also a stronger between neighbourhood association, A situation that was confirmed in Model 3 (see under “Specific contextual average effects”) since the neighbourhood income was, on average, strongly associated to choosing a private GP.

#### Specific contextual effects: IOR and POOR

Model 3 shows that high neighbourhood income was, on average, strongly associated with visiting a private physician (OR = 3.50). So the customary interpretation would be that, over and above individual income, age and sex, living in a high income neighbourhood strongly increased the individual probability of visiting a private physician. However, this contextual variable only explained a small share (PCV = 11%) of the initially large neighbourhood variance. Therefore, unmodeled variability between neighbourhoods remained large as expressed by the wide IOR-80% = 0.09 -.130.28 Also the POOR indicated that 33% of the time an individual from a high income neighbourhood had a lower, rather that higher, likelihood of visiting a private GP than an individual from a low income neighbourhood. That is, the average OR hides strong heterogeneity around the average association.

#### General Contextual Effects: Neighbourhood variance, ICC, MOR and AUC

**Fig 4:**
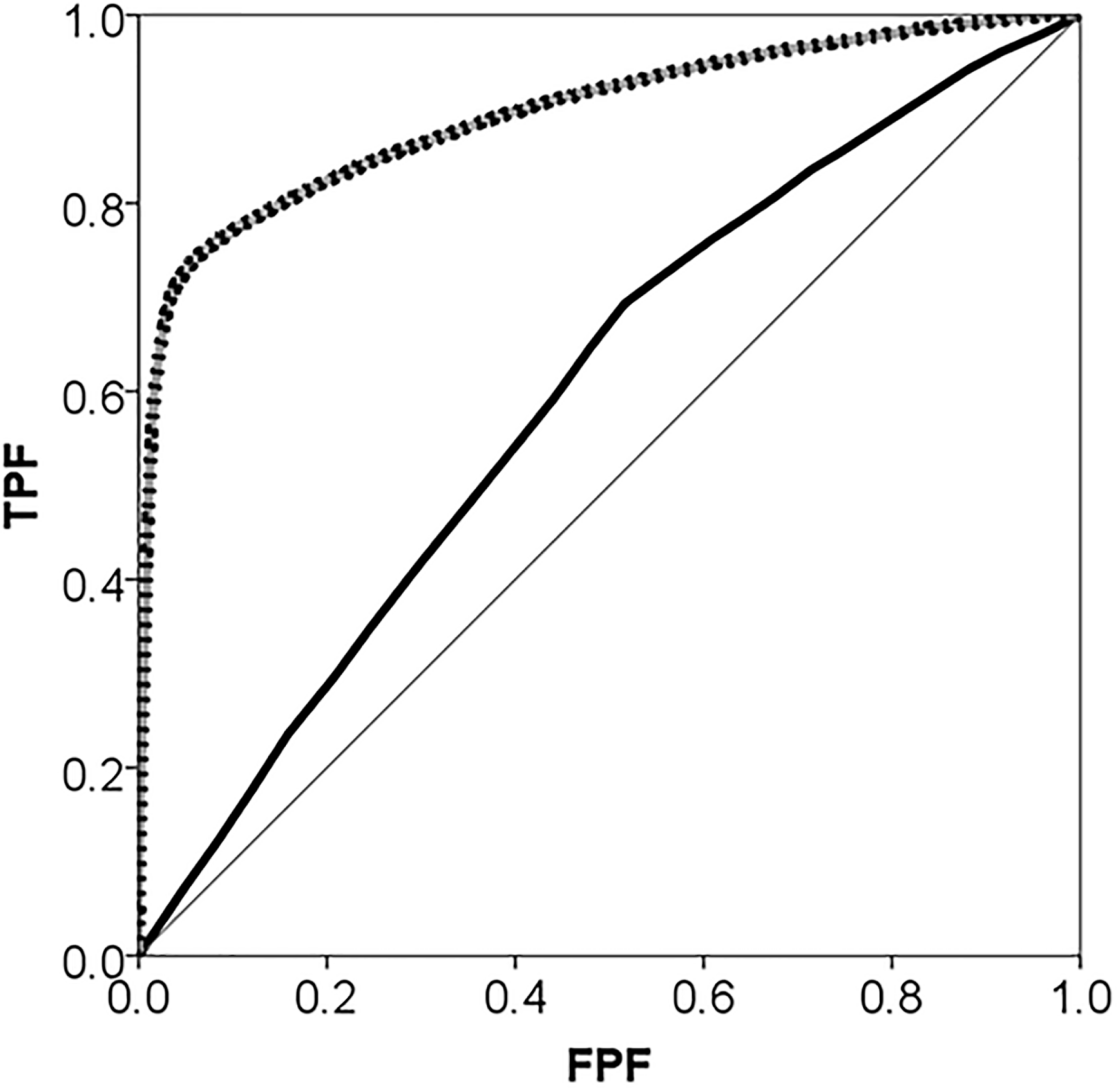
Areas under the receiver operating characteristic curve (AUC) for choosing a private vs. a public GP during 2006 in the city of Malmö, Sweden plotted separately for Model 1 which only adjusts for individual-level covariates age, gender and income (black thick line); and for Model 2 which additionally adjusts for the neighbourhood of residence (grey dotted line). The diagonal line represents an AUC equal to 0.50.

If the neighbourhood context were relevant for understanding individuals’ choice of private vs public GPs we would expect a high ICC, a high MOR and a high increase of the AUC in Model 2 compared to Model 1. This is just what we found. The ICC in Model 2 was close to 60% and the MOR close to 8 which are very high values in “neighbourhood and health” studies. Furthermore, adding information on neighbourhood in Model 2 increased the AUC of Model 1 from about 0.6 to almost 0.9 which indicates that knowing the neighbourhood of one’s residence allows us to predict with rather high accuracy if an individual will choose a private versus a public GP (see Fig 4).

If the large observed general neighbourhood effect were mediated by neighbourhood income (or by other unobserved neighbourhood characteristics that this covariate may proxy for) we would expect a considerable reduction of the neighbourhood variance, the ICC, and the MOR in Model 3 compared with Model 2. However, this was not the case. In support of this argument, measuring the AUC using only individual variables and neighbourhood income but not the neighbourhood random effect gave an AUC (95% confidence interval) = 0.620 (0.614–0.626) which is only 0.03 units higher that Model 1 with only individual level variables.

**Fig 5:**
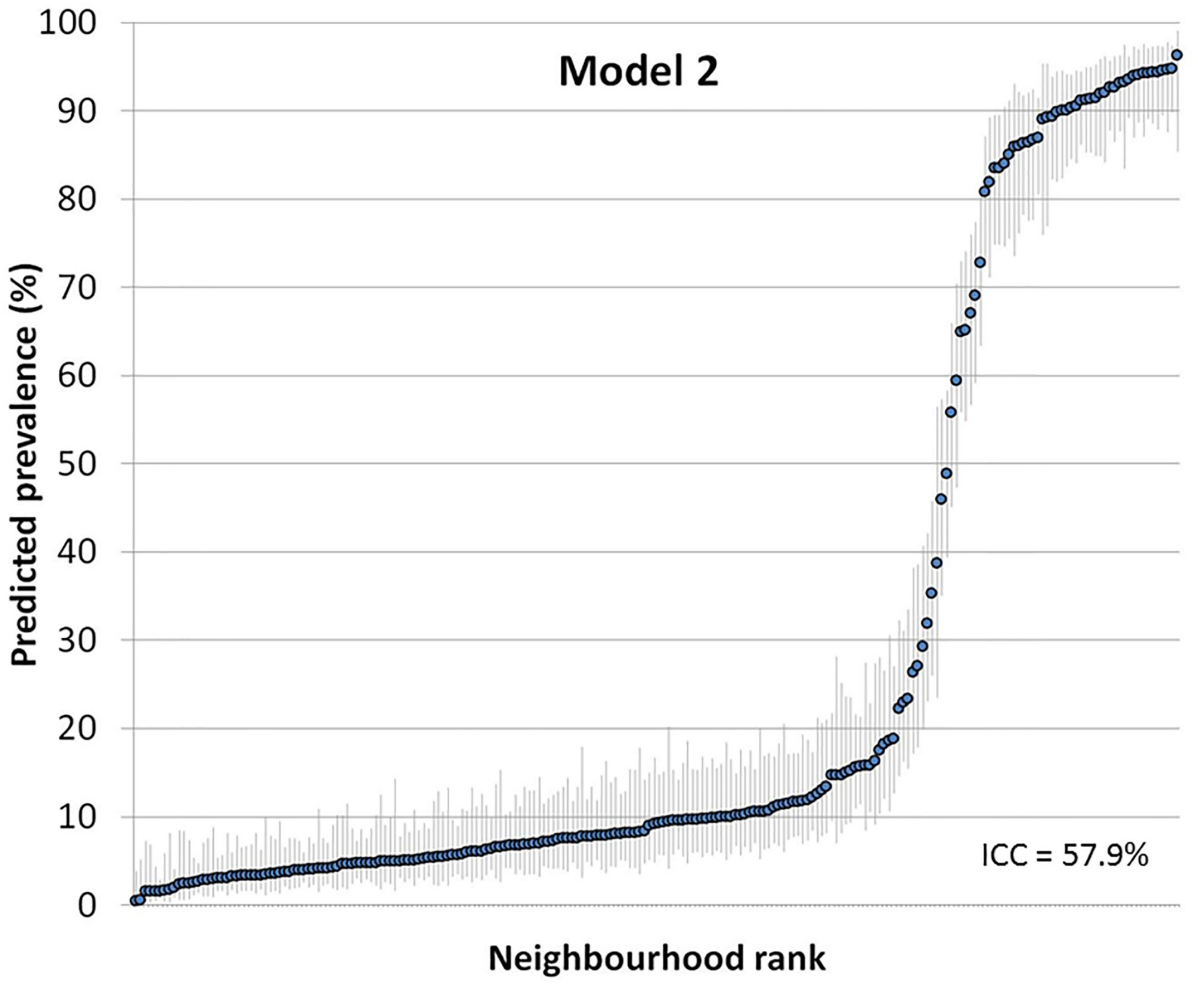
Predicted prevalence of using a private physician in each neighborhood with 95% confidence intervals versus ranking. Predictions are for the reference individual in model 2, i.e., female, age 35–39, low income.

**Fig 6:**
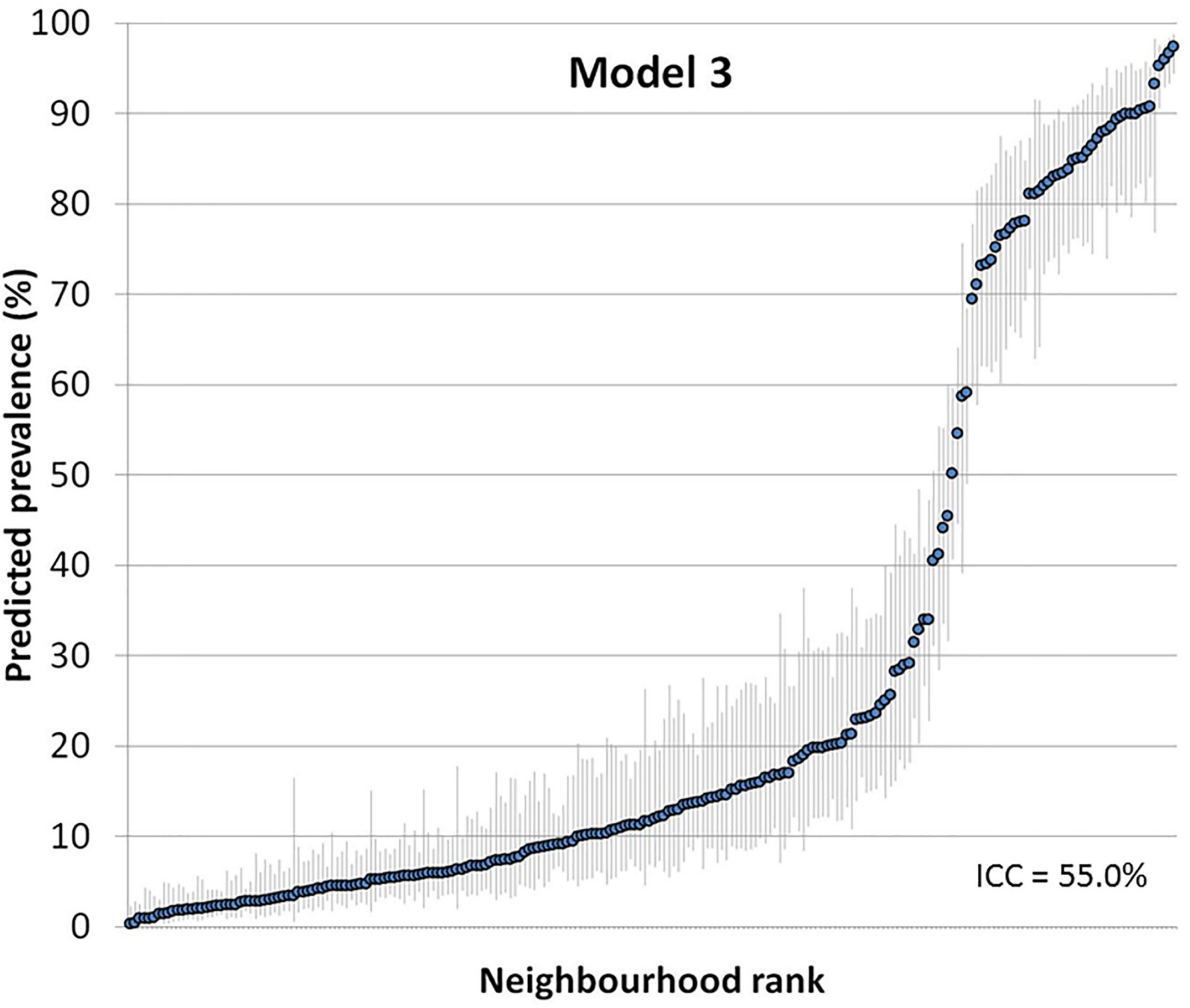
Predicted prevalence of using a private physician in each neighborhood with 95% confidence intervals versus ranking. Predictions are for the reference individual in model 3, i.e., female, age 35–39, low income and living in a low income neighborhood.

Figs 5 and 6 show the ranking of the neighbourhoods of Malmö in 2006 according to their predicted prevalence of using a private physician in each neighborhood with 95% confidence intervals versus ranking. Predictions are for the reference individual, i.e., female, age 35–39, low income (model 2) and also living in a low income neighborhood (model 3).

We observed a bimodal distribution for the neighbourhood differences with two groups of neighbourhoods, one smaller group with a higher probability of visiting a private GP, and another larger group with a lower probability. This bimodality reflects the underlying nature of private GP use. In our case, it revealed that over and above age, sex and individual income, individuals in some neighbourhoods mostly visit private physicians while individuals in other neighbourhoods mostly visit public GPs. A similar bimodality is frequently observed when there are strong general contextual effects in other settings as is the case when analysing individual within households [4, 49], sibling within families [50], or children within mothers [51].

This bimodality was not a concern for the statistical analysis as the number of neighbourhoods was high, which makes the assumption of normally distributed random effects less relevant [52]. Nevertheless, adjusting for neighbourhood income (low vs high) reduced the bimodality and it is assumable that the bimodality might be further reduced by modelling neighbourhood income in a more flexibly way (e.g., by entering a continuous measure of neighbourhood income as a polynomial). The pattern of neighbourhood differences also suggests the existence of spatial correlation which could be conditioned by the segregation of private practices in specific geographical areas. It is possible to allow for spatially correlated random effects in multilevel logistic regression, but this is beyond the scope of the current article.

We also note that there was high individual socioeconomic segregation. Multilevel logistic regression analyses have recently been proposed for modelling social and other forms of segregation [53–55]. Applying those ideas to our data, we fit a separate multilevel logistic regression analyses, modelling low individual income as the response variable. We estimated a neighbourhood variance of 1.032 which corresponds to an ICC of 24% and substantial segregation. Therefore, adjusting neighbourhood income for individual income is to some extent based on extrapolations since there are relatively few individuals with high income living in poor neighbourhoods and relatively few individuals with low income living in rich neighbourhoods.[56]

## Discussion

We have presented two applications illustrating how to use multilevel logistic regression analysis of heterogeneity to estimate individual and neighbourhood influences on individual health and health care utilization. Our three-step approach distinguishes between specific (measures of association) and general (measures of variance) contextual effects, and demonstrates the relevance of combining both approaches for gaining greater substantive understanding of the phenomenon under study. We analyse two different individual outcomes (psychotropic drug use and visit to a private vs. public GP) for which the relative importance of neighbourhood influences differs substantially. Our results agree with previous studies on the city of Malmö observing a large general neighbourhood effect for individual choice of private physician in 1999 (i.e., ICC = 33%, MOR = 3.36) [29] but a minor general neighbourhood effect for use of anxiolytic-hypnotic drugs (i.e., ICC = 1.7%, MOR = 1.25) in 1991–1996 [57].

We question the current probabilistic, risk factor epidemiological approach based on the simple interpretation of ORs for specific individual and contextual (e.g., neighbourhood) characteristics in isolation [2]. We promote a three-step multilevel analytical approach. Step 1 consists of fitting a single-level logistic regression adjusting for only the individual-level covariates, then evaluating the ORs and calculating the discriminatory accuracy (e.g., AUC) of these variables. Step 2 consists of extending the model to two-levels (by adding the neighbourhood random effect) and then assessing the importance of general contextual effects using the ICC and AUC. Step 3 consists of adding specific neighbourhood characteristics (i.e., specific neighbourhood effects) to the model and interpreting their ORs jointly with the size of the initial general contextual effect and the size of the neighbourhood variance explained (i.e., PCV). We argue that the incorrect population average interpretation of the OR for contextual variables needs be avoided. For this purpose the IOR or the POOR should be presented side-by-side with the average OR.

### Psychotropic drug use

Applying our three-step approach to psychotropic drug use, we observed that sex, increased age, and individual low income were associated with the use of this medication. However, the information provided by these individual characteristics did not allow users of psychotropic drugs to be distinguished from non-users with any degree of accuracy (AUC = 0.616). We also observed a very small general contextual effect since accounting for neighbourhood of residence only increased the AUC by 0.014 units and both the ICC (i.e., 1.1%) and the MOR (i.e., 1.20) were very low. In fact, our results suggest that SAMS neighbourhoods were more similar to simple random samples from the population of Malmö, than to meaningful contexts influencing individual psychotropic drug use.

The low AUC of the neighbourhood context (i.e., the low general contextual effects) needs to be considered when interpreting the small but conclusive association between low neighbourhood income and individual use of psychotropic drugs. One could argue that this neighbourhood variable explained 42% of the neighbourhood variance, but as such variance was rather small (i.e., ${\hat{\sigma }}_{u}^{2}$ = 0.038), it actually explained a lot of very little. Furthermore, the POOR informed that 11% of the time the positive association between low neighbourhood income and individual psychotropic drug use was in the opposite direction with a decreased, rather than increased, propensity of using psychotropic drugs in the low income neighbourhoods.

Paradoxically, when the neighbourhood variance is low (i.e., there is a weak general contextual effect) it is easier to obtain “significant” associations with narrow 95% CI for the contextual variables (i.e., specific contextual effect). This situation happens because we assign the values of neighbourhood variable to uncorrelated individuals in the sample. In other words, the less neighbourhood boundaries matter for the outcome, the easier it is to get “significant” associations between specific neighbourhood characteristics and the individual outcome. When researchers plan a study of “neighbourhood and health”, they typically assume that there is a strong intra-neighbourhood correlation. However, we need to check this assumption and always interpreted the specific contextual effect (i.e., OR and 95% confidence interval) considering the size of the initial general contextual effects (e.g., ICC or AUC). Following the three-stage approach promoted in this article ensures a more appropriate interpretation.

The low general neighbourhood effects could be related to the fact that psychotropic drug use may be conditioned by other kind of contexts like the physicians or the Primary Health Care centres where the individuals are treated. The SAMS areas were relatively easy to obtain but their definition was not based on robust theory related to the contextual processes and mechanisms that may condition use of psychotropic drugs (or, for that matter, the choice of a private GP). In fact, the relevant context may not be at the neighbourhood level at all. Prescription of psychotropic drugs is homogenously regulated all over Sweden [58], which may reduce the influence of the neighbourhood on individual use of this medication. However, larger contextual effects might be observed when studying countries with different health care systems and therapeutic traditions or where psychotropic drugs are available over the counter. We have previously observed such a situation in the context of studying blood pressure. We identified a very low general contextual effect of the city areas in Malmö [10], but this effect was much higher when analysing countries with different health care systems [11]

In summary, we were not able to identify with accuracy the factors that predict psychotropic drug use. What we did find was that individual age, sex, and low income appeared to be poor predictors for identifying users of psychotropic drugs, and additionally including neighbourhood of residence did not alter this situation. That is, the neighbourhood context had only a negligible influence on individual use of psychotropic drugs.

### Choice of a private vs. a public GP

Concerning individual choice of private vs. public GP, our analysis showed that while the sex of the individual was not related to this choice, age was weakly positively associated and individual high income strongly associated (OR = 2.13) to this choice. However, as in the case of psychotropic drug use, the low discriminatory accuracy (AUC = 0.600) rendered the information supplied by these individual-level covariates insufficient for distinguishing who would choose a private vs. public GP. However, we found a very strong general contextual effect since accounting for neighbourhood of residence in the analysis increased by 0.295 units the AUC to 0.895. Also, the large ICC (i.e., 57.8%) and MOR (i.e., 7.53) values indicate that SAMS neighbourhoods captured a meaningful context influencing this individual behaviour. The socioeconomic context of the neighbourhoods (i.e., high vs. low neighbourhood income) was, on average, associated with choosing a private GP (OR = 3.50). However, this specific neighbourhood variable only explained 11% of the large neighbourhood variance in Model 2 (i.e., ${\hat{\sigma }}_{u}^{2}$ = 4.479). In fact, in as much as 33% of comparisons between rich and poor neighbourhoods, the OR for neighbourhood income was in the opposite direction so high neighbourhood income was associated to a lower rather than a higher propensity of choosing a private GP.

In our analyses we used a rough dichotomization of area socioeconomic circumstances (i.e., rich vs poor areas). This dichotomization was motivated to reinsure the anonymity of the information but it may reduce the neighbourhood variance explained by area variable. However, in a separate analysis, categorizing this variable in decile groups the variance explained increased to 16%, which is not a radical change comparing with the original 11%. Some reader may consider 11% as an important percentage. Comparing the explanatory power of a neighbourhood covariate to that found for competing covariates in the same or similar studies may be the only sensible way to be able to discuss whether a neighbourhood variable is relevant. If binary neighbourhood covariates rarely explain high proportions of neighbourhood variance then 11% may well be considered high. However, we think that it is also necessary for researchers to consider the size of the initial general contextual effect since the binary covariate may well explain a lot of very little (i.e., a high proportion of a small absolute amount of neighbourhood variation). In our private physician example, however, the initial general contextual effect was substantively large and so 11% would then be considered both a high proportion and substantively meaningful in terms of the absolute variation it generates in the predicted probability of having a positive response. However, the fact remains that 89% of the neighbourhood variance still persits unexplained and is attributed, collectively, to other unmodeled factors. Whether any of these unobserved factors individually explains a much higher proportion of the neighbourhood variance we can only speculate.

We observed that, on average, utilization of private GPs was higher among high income people and in high income neighbourhoods than in the low income categories, which deserves a closer analysis. In fact, access to health care in Sweden is by law [59] on equal terms and according to needs, and for many years societal funding has equally financed both public and private health [60] so economic circumstances should not be the main reason for choosing a public vs. a private GP [60]. The observed link between income and utilization of private GPs might depend on cultural preferences rather than solely on economic reasons. It is known, for example, that choice of sector also carries a symbolic meaning [61] and high income individuals have been argued to intrinsically prefer private care. However, an alternative explanation could be the existence of “invisible” barriers like adverse attitudes of private GPs against low income individuals, which might channel those individuals towards public GPs [60].

In summary, neither the simple comparison of the proportion of individuals visiting a private GP in rich vs. poor areas (35% vs 11% respectively) nor the OR of the neighbourhood variable inform of the discriminatory accuracy of the income variable. Rather the multilevel analysis including the AUC as a measure of discriminatory accuracy informed that over and above individual characteristics the neighbourhood of residence strongly predicted the choice of a private vs. a public GP, but the reasons for this phenomenon are only partially explained by socioeconomic circumstances of the neighbourhoods. On average, individuals residing in high income neighbourhoods had a higher propensity of visiting a private GP, but this contextual variable only explained a low proportion of the variation in neighbourhood differences. Other contextual factors not considered in our analysis, for instance, the degree of private GP provision in each neighbourhood might go some way to explaining the observed general contextual effects.

### Public Health implications

According to our analyses, policies to improve psychological health or reduce the use of psychotropic drugs in the city of Malmö would need to realize that focusing on specific neighbourhoods would not be effective because of the low discriminatory accuracy of this information. In fact, the same is true for the individual characteristics we analysed: age, sex, and income. Put differently, neither neighbourhood of residence nor the individual characteristics studied provided accurate information for identifying target groups. If policy makers do choose to focus on those individuals and neighbourhood with a higher average risk of using psychotropic drugs (which would be the normal procedure in risk factors epidemiology), they need to be aware that many psychotropic users would be labelled as “low risk” and that many non-users of psychotropic drugs would be labelled as “high-risk”. That is, focusing on only high risk groups would unnecessarily expose many individuals to an intervention they do not need and would leave many individuals untreated because they belong to low risk groups. Perhaps a better approach would be to launch an intervention on the whole population. In any case, considering the balance between harms and benefits, an intervention with low discriminatory accuracy conveys that the principle of *primum non nocere* must be an absolute condition.

The public health implications of our second analysis are very different. Here, policies to increase the use of public GP services should mostly focus on specific neighbourhoods, perhaps by opening local public GP alternatives.

Our analyses are based on real data and provide useful information for understanding neighbourhood level influences in general and on individual use of psychotropic drugs and choice of GP in particular. However, our primary aim was to illustrate how to perform and interpret a multilevel analysis of individual heterogeneity in social epidemiology and public health so we only considered a few variables. In any case, our study shows that neighbourhood “effects” are not properly quantified by differences between neighbourhood averages but rather by measuring the share of the individual heterogeneity that exists at the neighbourhood level.

### Multilevel analysis of heterogeneity and risk factors epidemiology

The multilevel analysis of heterogeneity we present in our study is rather innovative [2]. Most studies analysing the role of individual or contextual variables on health adopt a probabilistic perspective based on the analysis of differences in average risk between exposed and unexposed groups [62] but without recognizing the value of analysing variance [63]. This is the classical approach in so called “risk factors epidemiology” and many multilevel analyses have only focused on the identification of contextual risk factors such as neighbourhood social capital and neighbourhood deprivation. From this perspective small or even tiny effects (e.g., OR = 1.5 or even lower) with very low discriminatory accuracy are considered relevant. The problem is that by doing so we promote population level policies and interventions that may lead to both under and overtreatment, as well as unnecessary side effects and costs. It also raises ethical concerns related to misleading risk communication and the perils of both unwarranted interventions and stigmatization of exposed individuals[2, 64].

The multilevel analytical approach we propose differs fundamentally from the classical one. First, we adopt a mechanistic perspective that tries to understand the individual heterogeneity of responses surrounding average probabilities. Second, we combine measures of association with measures of variance and discriminatory accuracy and stress the importance of evaluating not only the discriminatory accuracy of the individual level variables but also of the geographical boundaries used to define neighbourhoods in relation to the outcome under investigation. For this purpose what we denominated *general contextual effects* in multilevel regression analysis allows us to quantify the degree of clustering within neighbourhoods (i.e., the ICC) [7, 14] or, analogously, the discriminatory accuracy of using the boundaries of the neighbourhoods in the analysis (i.e., the AUC) [33, 34]. The existence of individual dependence within neighbourhoods is not only the *sine qua non* for applying statistical multilevel analyses but also the size of this dependence provides fundamental substantive information [2, 3].

### Strength and weaknesses

Our current study tries to quantify the relevance of neighbourhoods in Malmö for understanding individual use of psychotropic drugs and choice of private vs public GP. We considered the simplest possible multilevel structure of individual nested within neighbourhoods as this is the most common design in neighbourhood and health studies. However, to constrain the study of contextual effects to a single geographical level (e.g., SAMS areas) is certainly an extreme simplification [65]. Individuals are likely to be simultaneously affected by multiple contexts at different scales across time [66–70]. Besides, other kind of contexts like the household are normally disregarded in studies of neighbourhood and health [4] [49]. Nevertheless, the analytical approach we promote can be developed for more than two levels of analyses (e.g., individuals nested in households nested in neighbourhoods) [4] as well as for multiple membership and cross-classified multilevel structures (e.g., schools and neighbourhoods at different times in the life course) [2, 67, 71–73]. However, adopting a pragmatic rather than academic perspective, straightforward multilevel analysis of heterogeneity that only considers individuals nested in neighbourhoods provides a better basis for informed decisions in public health than the simple ecological or spatial analyses of small area variation or classical multilevel analysis of contextual risk factors [4].

The identification of causal effects in observational epidemiology and, more specifically, in the study of neighbourhoods “effects” is a major problem. In our study, the underlying causal question was to know what would happen to an individual if she/he, *ceteris paribus*, moves to another neighbourhood with a different context. Furthermore, we wanted to identify if any general effect was mediated by a specific variable informing the socioeconomic characteristics of the context (e.g., rich vs. poor neighbourhood). However, what “rich” and “poor” neighbourhood means is difficult to specify and it would need a deeper sociological analysis. In the adjusted analysis we only considered individual age, sex and income as our main purpose was to illustrate the methodology. Therefore, we cannot exclude the existence of omitted confounding factors. Nevertheless, in neighbourhood analyses it is always a caveat to distinguish between confounder and mediator variables as frequently a common cause of both place of residence and the health outcome may also be a mediator of the neighbourhood effect (for instance low income is associated to using psychotropic drugs and low income individuals may be segregated to poor neighbourhoods but, in turn, living in a poor neighbourhood may reduce the chances of increasing an individual’s income). Furthermore, there may be problems of extrapolation (i.e., making inferences beyond the range of the data,) since few rich individuals reside in poor neighbourhoods and vice versa, so the appropriateness of adjusting for individual income could be questioned. Finally, while some contextual effects may be caused by exogenous exposures (e.g., absence of public GPs in an area) other may be endogenous and emerge from the individual composition of the neighbourhood (e.g., switching all low and high income individuals to rich and low neighbourhood will also change the neighbourhood context). In general, drawing valid causal inferences in observational epidemiology is difficult and this is especially the case in neighbourhood and health studies [2, 56].

### Correspondence between the different measures used to estimate general contextual effects

When using model predictions that include a random intercept [74, 75], as in the present study, there is a clear correspondence between the ICC and the AUC. That is, when the ICC is high the AUC is also high. However, under certain circumstances like when the number of individuals is relatively much larger in some neighbourhood than in others and/or if the number of neighbourhood is low, it could be possible to find a discrepancy between the ICC and the AUC (for instance the ICC may be high but the AUC low). The reason for this discrepancy is that the calculation of the ICC is based on the neighbourhood variance which, in turn, is based on differences between neighbourhoods’ averages and it is, therefore, standardized for neighbourhood size (i.e., the number of individuals in the neighbourhoods). On the other hand, the AUC is based on the calculation of the TPF and FPF for different thresholds of the predicted probability. Since this predicted probability is an individual level variable, large clusters contribute with more individuals. In other words, the AUC measure is a weighted average of a within- and a between-cluster part of the individual predictions, where the ICC measure is based on the neighbourhood variance (i.e., random effects) only [75]. This situation does not mean that the AUC is a biased measure but, rather, it provides different and useful information. For instance, some large neighbourhoods could have a *high* proportion of individuals visiting a private GP and some small neighbourhoods could have a *low* proportion of individuals visiting a private GP. The ICC would be high indicating that neighbourhoods condition the individual choice of private versus public GP. However, the AUC would be low expressing that most individuals have the same predicted risk, irrespective of whether they visit a private GP or not, and subsequently, that the neighbourhoods do not discriminate with accuracy individuals that visit a private GP from those who do not. Otherwise, when neighbourhoods sizes are similar and the number of clusters is large—as in the present study—there is a clear correspondence between the ICC and the AUC values [33, 34]. A more detailed description of the different predictions and measures in a multi-level setting is given by Van Oirbeek and Lesaffre [75]

In summary, in many cases, when the clusters (e.g., neighbourhoods) are of similar sizes as in our study, there is a good correspondence between the AUC and the ICC. However in certain situations when some neighbourhoods are of different sizes the ICC and the AUC may provide complementary information. This is an innovative concept that needs be further developed [34].

There is also a correspondence between the MOR and the ICC as both are monotone functions of the neighbourhood variance, and this correspondence makes the MOR a measure of general contextual effects. However, the MOR is a measure of probability and not of components of variance as the ICC. The MOR expresses the size of the heterogeneity between the neighbourhoods and the ICC the size of the clustering within neighbourhoods.

### The identification of the units of analysis

In contextual epidemiology the individual units are obviously easy to recognize since each individual is delineated by the skin. However, this is not the case when it comes to identifying contextual units. For this purpose, we frequently use geographical and administrative boundaries delineating small areas such as neighbourhoods, blocks, census tracts, or even large territories such as states, counties or countries. We assume that these boundaries condition individual health over and above individual characteristics. Nevertheless, this assumption is rarely validated [7]. The components of variance analysis and the use of measures of discriminatory accuracy help us to identify if the definition of neighbourhood we use actually captures a relevant context that influences the health outcome under investigation. Different neighbourhood definitions clearly have different relevance for the same outcome while the same neighbourhood definition may have different relevance for different outcomes.

### The “fixed effects” approach for the calculation of the neighbourhood AUC

An alternative to the use of the predicted neighbourhood random effects for the calculation of the AUC is to include the neighbourhoods as fixed-effects dummy variables in a single level logistic regression (i.e., “fixed effects approach”). Using this alternative we obtained an AUC (95% confidence interval) equal to 0.895 (0.891–0.899) for visiting a private vs a public GP, and equal to 0.634 (0.628–0.640) for use of psychotropic drugs which are very similar to those obtained from Model 2 (general contextual effects) of the multilevel regression analysis. The “fixed effects approach” provides a worthy strategy for a quick evaluation of general contextual effects and it does not require special software for multilevel analyses. However, the fixed effects approach prevents the further study of contextual level variables (e.g., neighbourhood low income). Besides, the model is not parsimonious. For instance in our study we would need to include 217 dummy variables for the 218 neighbourhood’s. The fixed effects approach is also susceptible to biased estimation by random noise if the number of individuals in some neighbourhood’s is small, while the prediction of neighbourhood effects in multilevel regression is based on empirical Bayes prediction which protects against this bias by being a so-called shrinkage estimator [16]. More fundamentally, the “fixed-effects” logistic model provides inconsistent estimates of the regression coefficients when the number of individuals per neighbourhood is low due to what is known as the *incidental parameter problem* [76] in which case it may be more appropriate to consider conditional logistic regression. Also, when the neighbourhoods are small the sampling error variance is large and therefore the fixed effects overestimate AUC values. On the other hand, when the neighbourhoods are small the shrinkage estimator of the random effects model will underestimate the AUC value. However, the random effects model outperforms the fixed effects model on smaller cluster samples, at least in terms of mean squared error and for this reason is preferable.

In summary, we think that the fixed-effects approach has a number of disadvantages and it is not better than the random effects approach for calculating the AUC. However, as the cluster size increases, the predicted cluster random- and fixed-effects will approach one another. As a result the AUC statistics based on each model will also converge.

### Empirical Bayes methods to estimate the random effects versus fully Bayesian using MCMC

Observe that we fitted the models by a fully Bayesian approach using MCMC. Therefore, we should have strictly reported the means of the MCMC chains for each predicted neighbourhood random effect. However, since the results are very similar, we simply predicted the neighbourhood random effects post-estimation using the model parameter estimates and data in the way one usually does when one has fitted the model by maximum likelihood estimation (i.e., the empirical Bayes prediction). The fully Bayesian procedure is so far technically more complicated and time demanding but can be performed following the indication provided in the section 4.6 of the MLwiN MCMC manual [48]. The advanced reader may apply the fully Bayesian procedure using the databases supplied as complementary information.

### Conclusions

In observational epidemiology of neighbourhoods and health, there are many unsolved problems concerning the identification of the relevant contexts for specific health outcomes. There are also specific difficulties for drawing causal inferences. Furthermore, in common with other fields in epidemiology, the traditional approach in multilevel analysis of neighbourhood and health maintains a probabilistic approach focused on the analyses of associations and considers the analyses of variance as a secondary task [63]. However, some authors, including ourselves [4, 9, 11, 12, 29, 66, 67, 69, 70, 77, 78] stress that the simultaneous consideration of both measures of association and of variance is fundamental in epidemiology [2]. The present study clearly illustrates that the bare analysis of measures of association is insufficient for understanding contextual effects on individual health. In fact, naïve interpretations of measures of associations and considering only the statistical “significance” of the neighbourhood variance is misleading and gives an inappropriate base for decision makers. Our study provides concepts and innovative analytical approaches like the use of the AUC that allow improved multilevel analysis of neighbourhood and health. Nevertheless, our study could be further developed by considering specific and general cross-level interactions. That is, the possibility that the individual level associations might be modified by the general neighbourhood context where the individual resides (i.e., random slope analysis), or by some specific contextual characteristics [17]. We need also to consider that the neighbourhood variance may be a function of the individual level variables. We encourage researchers to explore those aspects in their own work. Also, rather than simple hierarchical multilevel structures we should considered multiple memberships cross-classified multilevel analyses [71] [73]. A relevant area of development is to integrate the concepts of *intersectionality* [79], *interaction analysis* [80] and advanced *multilevel analysis of individual heterogeneity* [2]. These different terms from different scientific traditions express the same concept in different languages and stress the idea of going away from simple measures of differences between group averages in order to understand population and individual heterogeneity. This concept is also embraced by modern systems science as complex systems are intersectional and multilevel [81].

Finally, performing and interpreting multilevel regression analyses is an interesting task and many technical and conceptual advances have been performed during the last three decades. However, in the end, the quantitative analysis of contextual influences on individual health may well be unsatisfactory no matter how sophisticated the statistical techniques. Public health would benefit from a stronger humanistic approach that combines multilevel regression and qualitative analyses [22, 82, 83]. In any case, epidemiological studies should always provide measures of discriminatory accuracy like the AUC side by side with measures of association.

## Supporting Information

S1 Calculation Sheet Excel sheet for the calculation of the Median Odds Ratio (MOR), 80% Interval Odds Ratio (80% IOR), Proportion of Odds Ratios of opposite direction (POOR), Intraclass Correlation Coefficient (ICC) and the Proportional Change of the Variance (PCV). (XLS)

S1 Data Fully anonymized database in MLwiN format. (WSZ)

S2 Data Fully anonymized database in SPSS format. (SAV)

S3 Data Fully anonymized database in Stata format. (DTA)

S1 Model MLwiN worksheet for running the equation of model 1 concerning utilization of a private versus public general practitioner. (WSZ)

S2 Model MLwiN worksheet for running the equation of model 2 concerning utilization of a private versus public general practitioner. (WSZ)

S3 Model MLwiN worksheet for running the equation of model 3 concerning utilization of a private versus public general practitioner. (WSZ)

S4 Model MLwiN worksheet for running the equation of model 1 concerning utilization of psychotropic medication. (WSZ)

S5 Model MLwiN worksheet for running the equation of model 2 concerning utilization of psychotropic medication. (WSZ)

S6 Model MLwiN worksheet for running the equation of model 3 concerning utilization of psychotropic medication. (WSZ)

S1 Stata Stata do-file for running all the models. (DO)
